# Prioritization approaches in the development of health practice guidelines: a systematic review

**DOI:** 10.1186/s12913-019-4567-2

**Published:** 2019-10-15

**Authors:** Amena El-Harakeh, Rami Z. Morsi, Racha Fadlallah, Lama Bou-Karroum, Tamara Lotfi, Elie A. Akl

**Affiliations:** 1 Center for Systematic Reviews on Health Policy and Systems Research (SPARK), American University of Beirut, Beirut, Lebanon; 2 Clinical Research Institute (CRI), American University of Beirut Medical Center, Beirut, Lebanon; 3 Faculty of Medicine, American University of Beirut, Beirut, Lebanon; 4 Department of Health Management and Policy, Faculty of Health Sciences, American University of Beirut, Beirut, Lebanon; 5 Global Evidence Synthesis Initiative (GESI) Secretariat, American University of Beirut, Beirut, Lebanon; 6 Department of Health Research Methods, Evidence, and Impact (HEI), McMaster University, Hamilton, Canada; 7 Department of Internal Medicine, American University of Beirut Medical Center, P.O. Box: 11-0236, Riad-El-Solh Beirut 1107 2020, Beirut, Lebanon

**Keywords:** Guidelines, Methodology, Priority setting, Prioritization approaches, Health priorities, Research prioritization, Guideline development

## Abstract

**Background:**

Given the considerable efforts and resources required to develop practice guidelines, developers need to prioritize what topics and questions to address. This study aims to identify and describe prioritization approaches in the development of clinical, public health, or health systems guidelines.

**Methods:**

We searched Medline and CINAHL electronic databases in addition to Google Scholar. We included papers describing prioritization approaches in sufficient detail allowing for reproducibility. We synthesized findings in a semi-quantitative way. We followed an iterative process to develop a common framework of prioritization criteria that captures all of the criteria reported by each included study.

**Results:**

Our search captured 33,339 unique citations out of which we identified 10 papers reporting prioritization approaches for guideline development. All of the identified approaches focused on prioritizing guideline topics but none on prioritizing recommendation questions or outcomes. The two most frequently reported steps of the development process for these approaches were reviewing the grey literature (9 out of 10, 90%) and engaging various stakeholders (9 out of 10, 90%). We derived a common framework of 20 prioritization criteria that can be used when prioritizing guideline topics. The most frequently reported criteria were the health burden of disease which was included in all of the approaches, practice variation (8 out of 10, 80%), and impact on health outcomes (7 out of 10, 70%). Two of the identified approaches stood out as being comprehensive and detailed.

**Conclusions:**

We described 10 prioritization approaches in the development of health practice guidelines. There is a need to assess the effectiveness, efficiency and transparency of the identified approaches and to develop standardized and validated priority setting tools.

## Background

The development of high-quality guidelines is a rigorous and complex process that requires an average of two to three years per guideline [1]. Due to the rapid accumulation of new evidence, guideline development should be followed by revisions and update as necessary [2]. Given the considerable efforts and resources required to develop and update guidelines, developers need to prioritize what topics and questions to address [3]. Therefore, priority setting is a key aspect of developing health practice guidelines [4, 5].

Priority setting should incorporate the values of various stakeholders while responding to a fundamental challenge faced by all health systems, the allocation of finite resources [6, 7]. Prioritization of guideline topics will direct efforts and funds towards the most important health needs, and will ensure that guidelines are focused and of a proper scope. This represents a step toward enhancing the delivery of evidence-informed care and improving health outcomes.

In addition to prioritizing topics, the guideline development process entails the prioritization of questions and outcomes [5, 8]. Similarly, the adaptation of guidelines may require prioritizing which questions addressed in the original guidelines will be adapted [9]. Also, updating guidelines requires prioritization of which guidelines, guideline sections, or recommendations need to be updated [10].

Some investigators have provided general guidance on the prioritization of topics in guideline development by highlighting essential criteria or describing guiding principles [11]. Others have developed detailed tools and approaches for prioritizing topics in guideline development. For example, Schünemann et al. recommended nine steps for priority setting as part of their guideline development checklist [8].

While recognizing the need to outline the various prioritization approaches and highlight common themes, the aim of this study was to identify and describe prioritization approaches in the development of clinical, public health, or health systems guidelines.

## Methods

Our study design consisted of a systematic review of the health literature to identify prioritization approaches in the development of health practice guidelines. We followed a detailed methodology that we describe in the protocol included in Additional file 1. The project’s team included expertise in the fields of guideline development and priority setting.

### Eligibility criteria

- ▪ Paper type: We included papers of all types except for editorials, commentaries, correspondences, letters to editors, news, and abstracts. We excluded reviews but assessed all of the addressed approaches for potential eligibility.
- ▪ Scope: We included papers describing a prioritization approach in the de novo development, update or adaptation of health practice guidelines addressing clinical, public health, or health systems topics. The description of the approach should be thorough enough to allow for reproducibility (at least one section dedicated to that description). We excluded papers describing prioritization exercises conducted during guideline development without providing a detailed description of the process used to develop the prioritization approach. We also excluded papers describing individual prioritization items or criteria. In addition, we excluded papers where the focus of the prioritization approach was different from the guideline development process (e.g., prioritization of quality indicators derived from clinical guidelines).
- ▪ Setting: We included eligible papers irrespective of whether the setting was low-, middle- or high-income countries, or primary, secondary or tertiary healthcare facilities.

### Search strategy

We searched Medline and CINAHL electronic databases from their respective dates of inception until July 2019. We developed the search strategy with the help of an information specialist. The search combined various terms for health prioritization and included both medical subject headings (MeSH terms) and free-text words. We did not restrict the search to specific languages or dates. The detailed search strategy is provided in Additional file 2. We complemented the electronic databases search with the manual search of Google Scholar. We also screened the reference lists of included and other relevant papers and reviews to retrieve additional studies.

### Study selection

Teams of two reviewers screened in duplicate and independently all titles and abstracts of identified citations for potential eligibility. We retrieved the full texts for citations judged as potentially eligible by at least one of the two reviewers. Then, teams of two reviewers screened the full texts in duplicate and independently for potential eligibility. They resolved disagreements by discussion or with the help of a third reviewer (EAA) when consensus could not be reached. We used a standardized and pilot-tested screening form. We also conducted two rounds of calibration exercises before the screening process.

### Data abstraction

Two reviewers (AEH and RZM) abstracted data from eligible studies in duplicate and independently. They used a standardized and pilot-tested data abstraction form. Disagreements were resolved by discussion or with the help of a third reviewer (EAA). We conducted a calibration exercise to enhance the validity of the process.

We collected the following data from each included paper:

- ▪ General characteristics of the approaches for prioritizing guideline topics: authors; location; year of publication; lead entity; target audience; field (e.g., clinical, public health, or health systems); focus of prioritization (e.g., guideline topic, recommendation questions, or outcomes); and type of guideline development (update, adaptation or de novo development);
- ▪ Steps of the development process for the approach; we used the abstracted data to come up with a common categorization of the steps (e.g., literature review, consensus building, ranking of proposed prioritization criteria, pilot testing, primary research and stakeholder involvement);
- ▪ Aspects proposed to be addressed when prioritizing guideline topics; we used the abstracted data to come up with a common categorization of the aspects (e.g., guideline development steps at which prioritization should happen, steps for generating an initial list of topics, prioritization criteria, types of stakeholders to involve and method for involvement).

### Data synthesis

Due to the nature of data, we synthesized the findings in a semi-quantitative way. We used the abstracted data to come up with common categorizations of relevant concepts (e.g., prioritization aspects, generation of initial list of topics), using an iterative process of review and refinement. As part of this process, we analyzed the content of each study at least twice; once when drafting the initial categories, and after producing an advanced draft. We reported the results in both narrative and tabular formats.

In addition, we followed an iterative process of drafting and revision to create a common framework of prioritization criteria that captures all of the criteria reported by each included study. Subsequently, we attempted to match the reported criteria to those of the common framework (see Additional file 3). The iterative process included drafting an initial list of criteria by one of the researchers (AEH) based on an initial review of the criteria reported in the different included papers. Another researcher (EAA) verified the resulting list to improve the clarity and relevance of the proposed criteria and to evaluate the need for potentially merging, adding, or modifying criteria. Then, multiple meetings were held to refine the list of criteria through discussion and consensus. A third reviewer (RF) verified independently the proposed criteria against the ones reported by the included studies. This represented an opportunity to review the criteria, suggest refinements, avoid redundancy and propose new criteria. This was followed by a meeting to resolve disagreements through consensus and finalize the criteria list.

## Results

### Study selection

**Fig. 1:**
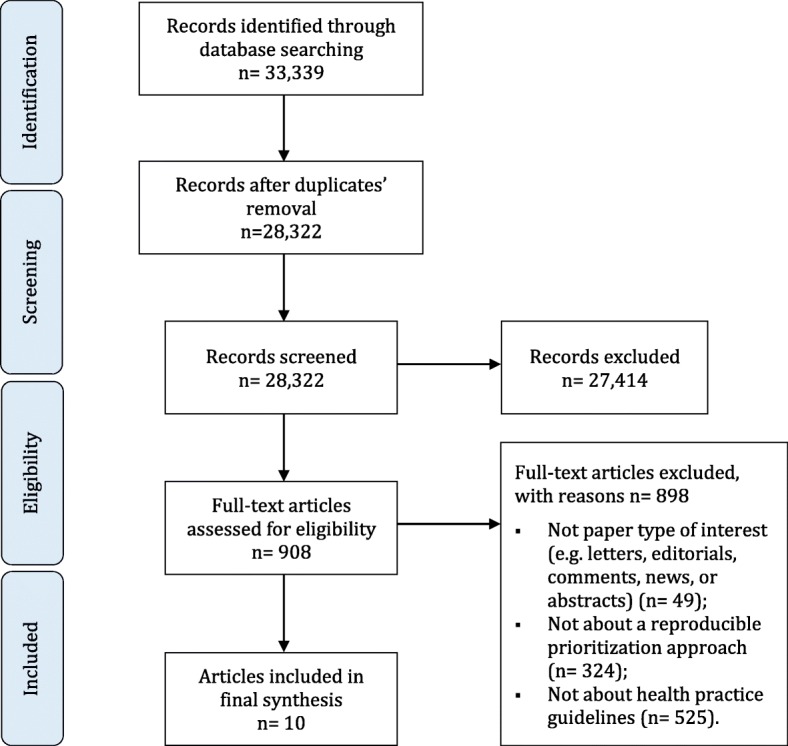
Preferred reporting items for systematic reviews and meta-analyses (PRISMA) study flow diagram for selection

Figure 1 shows the study flow diagram which summarizes the selection process. Out of the 33,339 citations identified through the electronic databases search, 10 papers met our inclusion criteria. We excluded 898 articles based at the full text screening stage for the following reasons: not paper type of interest (*n* = 49), not describing a prioritization approach (*n* = 324), not about practice guidelines (*n* = 525). We provide a detailed tabular description of each of the 10 included prioritization approaches in Additional file 4.

### General characteristics

The general characteristics of the 10 distinct approaches for prioritizing guideline topics described in the papers are reported in Table 1. Most of the papers (7 out of 10, 70%) focused on guidelines for clinical practice [12–14, 16–18, 20]; one paper proposed a prioritization approach that is applicable to the clinical, public health and health systems fields [8]; and the two remaining papers proposed prioritization approaches respectively for World Health Organization (WHO) healthcare recommendations [15] and for public health guidelines [19]. All of the identified prioritization approaches focused on prioritizing guideline topics; none on prioritizing guideline recommendation questions or outcomes. None of the approaches were specific to the update or adaptation of guidelines; all focused on the de novo development of practice guidelines.

**Table 1:** General characteristics of the approaches for prioritizing guideline topics

Paper	Lead entity	Target audience	Field (specific domain)	Focus of prioritization	Type of guideline development
Battista, 1995 [12]	Independent researchers	Canadian guideline developing groups	Clinical	Topics	De novo
Field, 1995 [13]	Institute of Medicine	Agency for Health Care Policy and Research	Clinical	Topics	De novo
McClarey, 1999 [14]	Royal College of Nursing (RCN)	RCN guideline developers	Clinical, nursing	Topics (e.g., hyperplasia, colon cancer, breast cancer, wound care, etc.)	De novo
Oxman, 2006 [15]	WHO Advisory Committee on Health Research	WHO entities developing guidelines	Health care	Topics or interventions	De novo
Ketola, 2007 [16]	‘Current Care’	Guideline developers	Clinical	Topics (e.g., benign prostatic hypertrophy, brain injuries in adults, atrial fibrillation, cataract, etc.)	De novo
Reveiz, 2010 [17]	Independent researchers	Guideline developers within developing countries	Clinical	Topics	De novo
Atkins, 2012 [18]	Independent researchers	Guideline developers in respiratory diseases	Clinical, respiratory diseases	Topics	De novo
Schünemann, 2014 [8]	Independent researchers	Guideline developers	Clinical, Public health and Health systems	Topics	De novo
Reddy, 2014 [19]	Independent researchers	National Institute for Health and Care Excellence (NICE)	Public health	Topic (e.g., sickle cell screening, substance misuse, water fluoridation, etc.)	De novo
Mounesan, 2016 [20]	Tehran University of Medical Sciences	Guideline developers	Clinical, family medicine	Topics (e.g., anemia, osteoporosis, indigestion/dyspepsia, pneumonia, etc.)	De novo

### Development process

Table 2 shows steps of the development process of each of the 10 included approaches for prioritizing guideline topics. The two steps most frequently reported to be used in the development process were: reviewing the grey literature (e.g., databases of guideline developing organizations) (9 out of 10, 90%) [8, 12–18, 20] and engaging various stakeholders (9 out of 10, 90%) [8, 12–17, 19, 20]. Patient and public involvement was reported to be used in the development of only one prioritization approach [14]. Conducting primary research was reported in the development of four out of the 10 approaches (40%) [12, 16, 17, 20]. The primary aim of conducting this type of research was to rate the importance of the suggested prioritization criteria and to assess the participants’ views regarding existing prioritization approaches in their respective organizations. Two studies followed all of the steps in the development process and were thus the most comprehensive and detailed [17, 20].

**Table 2:** Steps of the development process of the approaches for prioritizing guideline topics

Paper	Peer-reviewed literature	Grey literature	Consensus building	Ranking of proposed prioritization criteria	Pilot testing	Conducting primary research	Stakeholder involvement
% papers reporting the step	70%	90%	60%	50%	40%	40%	90%
Battista, 1995 [12]	✓	✓		✓		✓Mailed survey	✓
Field, 1995, 1995 [13]	✓	✓	✓				✓
McClarey, 1999 [14]		✓					✓
Oxman, 2006 [15]	✓	✓					✓
Ketola, 2007 [16]		✓	✓	✓	✓	✓Phone interviews	✓
Reveiz, 2010 [17]	✓	✓	✓	✓	✓	✓Online survey	✓
Atkins, 2012 [18]	✓	✓					
Schünemann, 2014 [8]	✓	✓	✓				✓
Reddy, 2014 [19]			✓	✓	✓		✓
Mounesan, 2016 [20]	✓	✓	✓	✓	✓	✓ Interviews	✓

### Aspects of prioritization

Table 3 shows the aspects proposed to be addressed when prioritizing guideline topics. Only one study highlighted the need to conduct prioritization during the various steps of guideline development, such as prioritizing the target audience, scope of guideline, questions of potential interest, effort of synthesizing evidence, recommendations, and recommendations for research [18]. Six studies (60%) included steps to generate an initial list of topics [8, 14, 16–18, 20]. Table 4 represents the steps proposed for generating an initial list of topics when prioritizing guideline topics. All of the studies incorporated the use of prioritization criteria as an aspect of the prioritization approach. Most of the studies (9 out of 10, 90%) included the involvement of stakeholders as one aspect of prioritization [8, 12–18, 20]. Table 5 shows the proposed types of stakeholders to involve in prioritizing guideline topics and the method for their involvement. Three studies covered the highest number of aspects of prioritization, that is four out of the five aspects [8, 18, 20].

**Table 3:** Aspects proposed to be addressed when prioritizing guideline topics

Paper	When to conduct prioritization?	How to generate an initial list of topics?	What criteria to use?	What stakeholders to involve?	Documentation
% papers reporting the aspect	10%	60%	100%	90%	40%
Battista, 1995 [12]			✓	✓	✓
Field, 1995 [13]			✓	✓	
McClarey, 1999 [14]		✓	✓	✓	
Oxman, 2006 [15]			✓	✓	✓
Ketola, 2007 [16]		✓	✓	✓	
Reveiz, 2010 [17]		✓	✓	✓	
Atkins, 2012 [18]	✓	✓	✓	✓	
Schünemann, 2014 [8]		✓	✓	✓	✓
Reddy, 2014 [19]			✓		
Mounesan, 2016 [20]		✓	✓	✓	✓

**Table 4:** Steps proposed for generating an initial list of topics when prioritizing guideline topics

Study	Description
Battista, 1995 [12]	Not reported
Field, 1995 [13]	Not reported
McClarey, 1999 [14]	1. Collect data using questionnaire from RCN professional groups and other RCN databases. 2. Collect information on patient priorities from representative groups and the literature. 3. Group topics by themes and accept that some might be arbitrary.
Oxman, 2006 [15]	Not reported
Ketola, 2007 [16]	1. Need for a new guideline arises in a specialist society or other source. 2. PRIO-tool from the ‘Current Care’ web site (http://www.kaypahoito.fi) is used to make a topic suggestion to the ‘Current Care’ board.
Reveiz, 2010 [17]	A thematic team (experts in the field and methodological consultant) would suggest three to five clinical topics that could potentially be selected for developing a clinical practice guideline.
Atkins, 2012 [18]	1. Survey clinicians, experts, and patients for candidate topics. 2. Create a list of topics using formal or informal (e.g., review of other guidelines). 3. Allow stakeholders to comment on scope and specific questions. 4. Identify issues arising from new and emerging technologies and treatments.
Schünemann, 2014 [8]	1. Decide who will oversee the process (e.g., priorities of the government, funding agency or professional society). 2. Apply specific criteria and use a transparent and systematic process to guide the suggestions of guideline topics.
Reddy, 2014 [19]	Not reported
Mounesan, 2016 [20]	1. Topic identification should be informed by evidence including: scientific evidence, available reports, expert opinion and/or needs assessment 2. Topic identification should be done separately for: prevention, diagnosis and treatment

**Table 5:** Common framework of the guideline topics prioritization criteria and their respective domains

Items	Disease-related factors					Interest			Practice		Guideline development				Potential impact of the intervention				Implementation considerations	
	Health burden	Economic burden	Burden on healthcare system	Equity relevance	Urgency	Health professional level	Consumer level	National level	Practice variation	Uncertainty or controversy about best practice	Absence of guidance	Unsatisfactory guidance	Availability of evidence	Potential for changing existing guidance	Impact on health outcomes	Economic impact	Impact on the healthcare system	Impact on equity/access	Feasibility of intervention implementation	Availability of resources
Paper																				
% papers reporting the criterion	100	50	30	50	10	40	40	20	80	40	50	50	50	50	70	50	40	20	40	30
Battista, 1995 [12]	✓												✓		✓	✓				
Field, 1995 [13]	✓	✓							✓						✓	✓				
McClarey, 1999 [14]	✓		✓	✓		✓			✓		✓		✓	✓	✓				✓	✓
Oxman, 2006 [15]	✓											✓		✓			✓		✓	✓
Ketola, 2007 [16]	✓	✓	✓	✓			✓		✓	✓	✓				✓	✓	✓			
Reveiz, 2010 [17]	✓	✓	✓	✓		✓	✓	✓	✓	✓	✓	✓	✓	✓	✓		✓	✓	✓	✓
Atkins, 2012 [18]	✓	✓				✓	✓		✓	✓	✓		✓		✓	✓	✓	✓		
Schünemann, 2014 [8]	✓	✓							✓	✓	✓	✓		✓						
Reddy, 2014 [19]	✓			✓					✓			✓	✓	✓						
Mounesan, 2016 [20]	✓			✓	✓	✓	✓	✓	✓			✓			✓	✓			✓	

### Prioritization criteria

We identified 118 prioritization criteria; 68% of the criteria (80 out of 118) were either defined or categorized under specific domains. 8% (9 out of 118) were supplied with data sources. The studies included a mean of 12 criteria (range 5–41). We derived from the 118 criteria a common framework of guideline prioritization criteria and of the domains they fall under. The framework is composed of 20 prioritization criteria clustered in six domains (Table 6) including: (1) disease-related factors; (2) interest; (3) practice; (4) guideline development; (5) potential impact of the intervention; and (6) implementation considerations. The most frequently reported criteria were related to the health burden of disease which was included in all of the prioritization approaches, practice variation (8 out of 10, 80%) [8, 13, 14, 16–20] and impact on health outcomes (7 out of 10, 70%) [12–14, 16–18, 20]. Urgency was included in only one of the approaches [20], while very few approaches reported criteria on an interest at the national level (2 out of 10, 20%) [17, 20] and on the potential impact of the intervention on equity/access (2 out of 10, 20%) [17, 18].

**Table 6:** Proposed types of stakeholders to involve in prioritizing guideline topics and the method for their involvement

Paper	Number	Involvement method	Type
Battista, 1995 [12]	Not reported	Not reported	• Members of guideline developing organizations • Potential end users • Patient representatives • Public
Field, 1995 [13]	Not reported	Delphi or Delphi-like techniques	• Experts • Potential end users (clinicians or patient representatives)
McClarey, 1999 [14]	Not reported	Not reported	• Professional guideline groups • Health care professionals • Patient representatives
Oxman, 2006 [15]	Not reported	Delphi technique	• Experts • Potential end users • Public • Others
Ketola, 2007 [16]	Not reported	Not reported	• Specialist society • Board members of guideline developing organization
Reveiz, 2010 [17]	> 12	Workshop, consensus meeting	• Experts • External guideline developers • Methodologist • End users
Atkins, 2012 [18]	Not reported	Not reported	• Clinicians • Professional organizations • Policymakers • Payers (e.g., health plans) • Government bodies • Quality organizations • Patient representatives
Schünemann, 2014 [8]	Not Reported	Not reported	• Clinicians • Professional societies • Policymakers • Payers • Public
Reddy, 2014 [19]	Not Reported	Not reported	Not reported
Mounesan, 2016 [20]	Range (5–15)	Face-to-face meeting	• Experienced family physicians • Management representatives

## Discussion

### Summary of findings

Our study aimed to identify and describe prioritization approaches that have been suggested in the development of health practice guidelines. We identified 10 prioritization approaches (seven for clinical practice, one for public health, one for WHO healthcare recommendations, and one for all three fields). There were variabilities in the steps followed to develop the approaches, in the aspects proposed to be addressed when prioritizing guideline topics, and in the prioritization criteria.

Stakeholder involvement and the use of prioritization criteria represented key aspects of most of the prioritization approaches. There is a global movement calling to increase the engagement of diverse stakeholders (consumers; health service providers; policy makers; and researchers) in developing research agendas and determining research priorities [21, 22]. The net benefit of this involvement needs to be further examined in developing prioritization approaches, as very few studies considered this aspect [23, 24].

We developed a common framework of prioritization criteria that captures all of the 118 criteria reported by the included studies. In the field of guideline development, recent documents on when and how to develop practice guidelines reported only examples of deciding which guidelines should be developed (e.g., WHO) [5].

A recent systematic review of the literature addressed prioritization but was limited to the update of health decision-making tools, one of which was guidelines [25]. Consistent with our findings, this systematic review found that the studies proposing an overall development strategy of guidelines did not provide a detailed description of the prioritization criteria used [25].

### Strengths and limitations

This study has several strengths. First, it responds to calls by researchers and health professionals globally emphasizing the importance of setting priorities in guideline development [26, 27]. To our knowledge, this is the first systematic review to describe prioritization approaches in the development of health practice guidelines. Another strength of the present study is that we used a rigorous and transparent process in its conduct (comprehensive search strategy, duplicate and independent selection, and duplicate and independent data abstraction) [28]. Finally, we followed an iterative process of drafting and revision to create a common framework of prioritization criteria that captures all of criteria reported by each of the ten included study. This represents a step towards standardizing the terminology for prioritization and enhancing the clarity of the criteria for decision-making.

One potential limitation of the study is that we did not search the grey literature and therefore we could have missed on potentially relevant information. However, we believe that our search was comprehensive enough and did not miss any study included by García et al. (2017) [25] that would have been eligible for our review.

## Conclusions

We described 10 approaches in the development of guidelines**.** The review findings can assist clinicians, funders, policymakers, and other stakeholders seeking to develop health practice guidelines in prioritizing topics to be addressed. It might be challenging to provide specific guidance on which approach to use given the variability in the processes followed to develop the approaches. However, guideline developers can choose the prioritization approach and criteria that best fit their needs.

The wide variability in the identified prioritization approaches necessitates that researchers develop standardized and validated priority setting tools in the development of health practice guidelines. There is also a need to develop methods for prioritization of questions and outcomes for guidelines projects. Researchers are encouraged to provide guidance on the conduct and reporting of studies on prioritization approaches.

Further rigorous methodological research is required to assess the effectiveness, efficiency and transparency of the identified approaches. This kind of evaluation research would lead to a better understanding of potential facilitators and barriers to prioritization. Furthermore, and because all of the included approaches were developed by researchers from middle- and high-income countries, future studies can focus on the effectiveness of the suggested approaches in low-income countries. It is also essential to evaluate the impact of those approaches on resource allocation and on clinical outcomes.

## Supplementary information

**Additional file 1.** Study protocol. The study protocol detailed the methodology of the systematic review.

**Additional file 2.** Search strategy. The search combined various terms for health prioritization and included both medical subject headings (MeSH terms) and free-text words.

**Additional file 3.** Framework of prioritization criteria in the development of health practice guidelines. The common framework of prioritization criteria captured all of the criteria reported by each included study.

**Additional file 4.** Detailed findings of the included papers on the development processes of the prioritization approaches and the aspects to be addressed when prioritizing guideline topics. This represents a detailed tabular description of each of the 10 included prioritization approaches.

## Data Availability

Not applicable.

## Abbreviations

MeSH terms: Medical subject headings
WHO: World Health Organization
