# Supplementary material for: Prioritization approaches in the development of health practice guidelines: a systematic review
Source: BMC Health Serv Res. 2019 Oct 15;19:692. doi: 10.1186/s12913-019-4567-2 (PMC6792189; doi:10.1186/s12913-019-4567-2)
Supplement: Supplementary file 2 — Additional file 2. Search strategy. The search combined various terms for health prioritization and included both medical subject headings (MeSH terms) and free-text words. [file 12913_2019_4567_MOESM2_ESM.docx]

# **Appendix 2:** Search strategy

**Databases Searched:**

| **Database** | **Number of hits** |
| --- | --- |
| Medline | 22,022 |
| CINAHL | 11,217 |
| Google Scholar | 100 |
| **Total** | **33,339** |

**Search Strategy for each database:**

**Database: Ovid MEDLINE(R)** Epub Ahead of Print, In-Process & Other Non-Indexed Citations, Ovid MEDLINE(R) Daily and Ovid MEDLINE(R) <1946 to July 2019>

Search Strategy (22,022):

--------------------------------------------------------------------------------

1 Methods/ (231687)

2 (framework or frameworks or approach or approaches or tool or tools or checklist or checklists or strategy or strategies or process or processes or criteria or toolkit or technique or techniques).ti,ab. (5817424)

3 1 or 2 (6021043)

4 Resource Allocation/ (8093)

5 health planning guidelines/ or health planning technical assistance/ or health priorities/ or regional health planning/ or health systems plans/ or community health planning/ (24610)

6 health care rationing/ (11219)

7 ("health planning" or "health priorit*" or "health systems plans " or "health care rationing" or "resource allocation").tw. (14849)

8 ((framework or frameworks or approach or approaches or tool or tools or checklist or checklists or process or processes or criteria or toolkit or technique or techniques) adj5 (priorit* or agenda? or (resource adj allocation))).ti,ab. (7306)

9 4 or 5 or 6 or 7 (52469)

10 3 and 9 (16501)

11 8 or 10 (22,022)

***************************

**Database: CINAHL**

Search Strategy (11,217):

--------------------------------------------------------------------------------

S1 1,097,726

MJ methods OR TI ((framework or frameworks or approach or approaches or tool or tools or checklist or checklists or strategy or strategies or process or processes or criteria or toolkit or technique or techniques)) OR AB ((framework or frameworks or approach or approaches or tool or tools or checklist or checklists or strategy or strategies or process or processes or criteria or toolkit or technique or techniques))

S2 21,343

MJ ( “Resource Allocation” OR “health planning guidelines” or “health planning technical assistance” or “health priorities” or “regional health planning” or “health systems plans” or “community health planning” OR “health care rationing” ) OR TX ( ("health planning" or "health priorit*" or "health systems plans " or "health care rationing" or "resource allocation") )

S3 (S1 AND S2) 6,625

S4 2,041

TI (((framework or frameworks or approach or approaches or tool or tools or checklist or checklists or process or processes or criteria or toolkit or technique or techniques) N5 (priorit* or agenda? or (resource N1 allocation)))) OR AB (((framework or frameworks or approach or approaches or tool or tools or checklist or checklists or process or processes or criteria or toolkit or technique or techniques) N5 (priorit* or agenda? or (resource N1 allocation))))

S5 (S3 OR S4) 11,217

***************************

**Google Scholar**

Last searched: July 2019

(method|framework|approach|tool|checklist|strategy|strategies|process|criteria|toolkit|technique) (health planning|health priority|health priorities|health prioritization|health systems plans|health care rationing|agenda|resource allocation)

*The 1^st^ 100 articles were retrieved
