# Supplementary material for: Prioritization approaches in the development of health practice guidelines: a systematic review
Source: BMC Health Serv Res. 2019 Oct 15;19:692. doi: 10.1186/s12913-019-4567-2 (PMC6792189; doi:10.1186/s12913-019-4567-2)
Supplement: Supplementary file 3 — Additional file 3. Framework of prioritization criteria in the development of health practice guidelines. The common framework of prioritization criteria captured all of the criteria reported by each included study. [file 12913_2019_4567_MOESM3_ESM.doc]

**Appendix file 3:** Framework of prioritization criteria in the development of health practice guidelines

# **Disease-related factors**

## Health burden

## Economic burden

## Burden on the healthcare system

## Equity relevance

## Urgency

# **Interest**

## Interest at the health professional level

## Interest at the consumer level

## Interest at the national level

# **Practice**

## Practice variation

## Uncertainty or controversy about best practice

# **Guidance development**

## Absence of guidance

## Unsatisfactory guidance

## Availability of evidence

## Potential for changing existing guidance

# **Potential impact of the intervention**

## Impact on health outcomes

## Economic impact

## Impact on the healthcare system

## Impact on equity/access

# **Implementation considerations**

## Availability of resources

## Feasibility of intervention implementation

| **Battista, 1995** | | |
| --- | --- | --- |
| **Authors’ prioritization criteria** | **Author’s prioritization themes** | **Prioritization criteria reclassification** |
| Are adequate data available? | Feasibility | Availability of evidence |
| What is the likelihood that the guidelines will make a difference in an outcome of interest (e.g., clinical practice, economic outcome or patients' quality of life)? |  | Impact on health outcomes; Economic impact |
| How many people have the condition for which the guidelines will be developed? | Population | Health burden |
| Will the guidelines improve health outcomes for them? |  | Impact on health outcomes |
| Are people with the condition likely to be affected by the guidelines? (i.e., Do people with condition X seek care from physicians who will be targeted for dissemination of guidelines for condition X?) |  | Impact on equity/access |
| Is the procedure or therapy expensive in unit (per case) terms or aggregate (all cases) terms? | Costs | Feasibility of intervention  implementation |
| What are the cost implications of the guidelines? (i.e., Will previously unmet needs increase aggregate costs? Will inappropriate care, and thus aggregate costs, be reduced?) |  | Economic impact |
| Will the guidelines likely result in improved quality of care and patient outcomes? | Effects on population, costs and practice | Impact on health outcomes |
| How will the costs of developing and implementing the guidelines compare with the projected costs (savings) accruing from their use? |  | Economic impact |
| Will the guidelines likely alter practice in a desirable way? What is the likelihood that developing and implementing the guidelines will have negative effects, either from the perspective of the developer or from other, perhaps contending, perspectives? |  | Impact on health outcomes |

| **Field, 1995** | | |
| --- | --- | --- |
| **Authors’ prioritization criteria** | **Authors’ examples** | **Final classification** |
| Prevalence of the clinical problem | number of affected persons per 1,000 persons in the general U.S. population | Health burden |
| Burden of illness imposed by the problem | individual mortality, morbidity, or functional impairment | Health burden |
| Cost of managing the problem | cost per person | Economic burden |
| Variability in practice | significant differences in utilization rates for prevention, diagnosis, or treatment options | Practice variation |
| Potential of a guideline or assessment to improve health outcomes | expected effect on health outcomes | Impact on health outcomes |
| Potential of guideline or assessment to reduce costs | expected effect on costs (to sponsoring organization, other relevant agencies, patients and families, and/or society in general) | Economic impact |

| **McClarey, 1999** | | |
| --- | --- | --- |
| **Authors’ criterion** |  | **Prioritization criteria reclassification** |
| Is the topic an area of care with excessive morbidity, disability or mortality? |  | Health burden |
| Do available treatments have established potential? |  | Impact on health outcomes |
| Is there wide variation in practice? |  | Practice variation |
| Is the service resource intensive? |  | Availability of resources |
| Is the topic one of high cost/low volume or high volume /low cost? |  | Burden on the healthcare system |
| Are there many boundary issues? |  | Equity relevance; Feasibility of intervention implementation |
| Is there already an existing guideline for the topic? |  | Absence of guidance |
| What work by other professional organizations is already addressing the topic? |  | Absence of guidance |
| Is there sufficient good quality research evidence available, preferably systematic reviews, to provide evidence for the guideline, or are there existing guidelines which could be updated? |  | Availability of evidence |
| Is there any information available about patient­ based outcomes and the impact the topic would have on patient care? |  | Potential for changing existing guidance |
| What topic would have the greatest impact on the nursing contribution to patient care? |  | Interest at the health professional level |

| **Oxman, 2006** | | |
| --- | --- | --- |
| **Authors’ criterion** |  | **Prioritization criteria reclassification** |
| Problems associated with a high burden of illness in low and middle-income countries, or new and emerging diseases. |  | Health burden |
| No existing recommendations of good quality. |  | Unsatisfactory guidance |
| The feasibility of developing recommendations that will improve health outcomes, reduce inequities or reduce unnecessary costs if they are implemented. |  | Potential for changing existing guidance |
| Implementation is feasible, will not exhaustively use available resources, and barriers to change are not likely to be so high that they cannot be overcome. |  | Availability of resources; Feasibility of intervention implementation |
| Interventions that will likely require system changes and interventions where there might be a conflict in choices between individual and societal perspectives. |  | Impact on the healthcare system; Feasibility of intervention implementation |

| **Ketola, 2007** | | |
| --- | --- | --- |
| **Authors’ criterion** | **Authors’ definitions** | **Prioritization criteria reclassification** |
| Frequency of the health problem | Incidence; Prevalence | Health burden |
| Extent of burden on the health care system | Number of visits; Number of procedures; Overlapping treatment periods in various specialties; Need for special training or special devices; Interest or demand from the population; Frequency of unnecessary examinations or treatments | Burden on the healthcare system; Interest at the consumer level |
| Economic effects on the health care system (estimated extent of change) | Costs of diagnosis and treatment; Expensive individual treatments or investments | Economic impact |
| Social effects (Absence from work; Disability to work; Retirement; Changes in the division of labor between professional groups; Need for institutional or informal care) | Absence from work; Disability to work; Retirement; Changes in the division of labor between professional groups; Need for institutional or informal care | Economic burden; Impact on the healthcare system |
| Variation of treatment practices | Use of different methods; Benchmarking; Schools of practice; Inequality in access to care; Regional variation | Practice variation; Equity relevance |
| Possibilities for health promotion and disease prevention | Prevention on a population level or among high-risk individuals; Lifestyle choices; Effect on quality-weighted years of life | Impact on health outcomes |
| Effectiveness and adverse effects of treatment | Availability of effective methods; Possibility of serious adverse effects; Treatment-induced effect on quality-weighted years of life | Impact on health outcomes |
| Need for information in health care | Contradicting information; New types of methods available; Discussion about values needed | Absence of guidance; Uncertainty or controversy about best practice |

| **Reveiz, 2010** | | |
| --- | --- | --- |
| **Authors’ prioritization criteria** | **Authors’ domains** | **Prioritization criteria reclassification** |
| Disease/Condition incidence or prevalence | Disease burden | Health burden |
| High risk impact of disease/condition in the health system |  | Burden on the healthcare system |
| High frequency of risk factors associated with the disease/condition |  | Health burden |
| High frequency of avoidable risk factors associated with the disease/condition |  | Health burden |
| Information needs within the Institution/ Organization | Information needs in the Health Sector | Absence of guidance |
| Current controversy about topic importance |  | Uncertainty or controversy about best practice |
| High importance of new methods and technology assessment |  | Potential for changing existing guidance |
| Fast diffusion of non-assessed technologies, availability of resources and sufficient time for technologies implementation |  | Absence of guidance; Availability of resources; Feasibility of intervention implementation |
| Country health priorities in agreement with CPG’s needs |  | Interest at the national level |
| High impact on national health system |  | Impact on the healthcare system |
| Feasibility on recommendations development which will improve health outcomes and cost | Feasibility on development and implementation | Potential for changing existing guidance |
| Is the proposal politically feasible? |  | Interest at the national level; Interest at the consumer level; Interest at the health professional level |
| Does it belong to priority health areas according to government policies? |  | Interest at the national level |
| Feasibility in implementation; will not require an excessive amount of resources and will not present important barriers to implement changes |  | Availability of resources; Feasibility of intervention implementation |
| will reduce inequities when implemented |  | Impact on equity/access |
| will require education to training professionals |  | Feasibility of intervention implementation |
| Does the proposed topic include the participation of multiple departments, institutions, organizations, etc.? |  | Feasibility of intervention implementation |
| Availability of effective methods shown by methodologically adequate studies. | Effectiveness | Potential for changing existing guidance |
| Certainty about effectiveness of assessed interventions and technologies |  | Potential for changing existing guidance |
| Potential impact of CPG |  | Impact on health outcomes |
| Economic effects on health system (cost of an individual patient is high during diagnosis or therapeutic process) | Economic impact on the health | Economic burden |
| Disease/condition associated with iatrogenic interventions that are significantly high in cost | system | Economic burden |
| Current evidence is insufficient for disease control in the population | Clinical Practice Variation | Unsatisfactory guidance |
| Lack of High quality CPGs |  | Unsatisfactory guidance |
| Availability of high volume of evidence regarding the CPG topic |  | Availability of evidence |
| Evidence of inappropriate use of available technologies used in the treatment of condition (iatrogenic) |  | Practice variation |
| Conditions/diseases where effective treatments could reduce mortality or morbidity |  | Impact on health outcomes |
| Evidence of disagreements between current treatment and literature recommendations. |  | Practice variation |
| Absenteeism from work or school, inability to work, inequities in access to health services | Other social effects/Equity | Economic burden; Equity relevance |
| Will the service be available to anyone who requires it? |  | Impact on equity/access |
| Will this CPG have a positive or negative impact on minorities’ access to health services? |  | Impact on equity/access |
| Will the CPG increase health service access to those affected by the condition? |  | Impact on equity/access |
| High patient demand or interest | User Preferences | Interest at the consumer level |
| Concerns about patients’ quality of life |  | Impact on health outcomes |
| Feasibility of patient empowerment |  | Feasibility of intervention implementation |
| High acceptability of the topic between the general public and professionals affected by the use of the CPG. |  | Interest at the consumer level; Interest at the health professionals level |
| Possibility of adverse events | Adverse events | Impact on health outcomes |
| Possibility of serious adverse events |  | Impact on health outcomes |
| Disease/condition associated with high incidence of adverse events or sequels |  | Health burden |
| Feasibility of prevention between patients with risk factors | Health Promotion and Disease Prevention | Impact on health outcomes |
| Are there specific activities of health promotion, disease prevention, early diagnosis or treatment? Have all of them shown a reduction in disease burden? |  | Impact on health outcomes |

| **Atkins, 2012** | | |
| --- | --- | --- |
| **Authors’ prioritization criteria** | **Authors’ data sources** | **Prioritization criteria reclassification** |
| Burden of disease (health or economic) | National data; review articles | Health burden; Economic burden |
| Costs of care | National data; review articles | Economic burden |
| Variability in practice | Quality measurement; surveys; expert opinion | Practice variation |
| Potential impact of guideline or recommendation | Expert opinion and stakeholder input | Impact on health outcomes; Economic impact; Impact on the healthcare; system Impact on equity/access |
| Importance to clinicians | Survey, consultation, and ad hoc stakeholder input | Interest at the health professional level |
| Importance to patients | Survey, consultation, and ad hoc stakeholder input | Interest at the consumer level |
| Availability of evidence | Existing reviews; preliminary literature search | Availability of evidence |
| Uncertainty or controversy | Literature search for editorials | Uncertainty or controversy about best practice |
| Emerging issues | Meetings, drug/device approvals, policy experts | Absence of guidance |

| **Schünemann, 2014** | | |
| --- | --- | --- |
| **Authors’ prioritization criteria** |  | **Prioritization criteria reclassification** |
| High prevalence and burden of disease |  | Health burden |
| Avoidable mortality and morbidity |  | Health burden |
| High cost |  | Economic burden |
| Emerging diseases or emerging care options |  | Absence of guidance |
| Variation in clinical practice |  | Practice variation |
| Rapidly changing evidence |  | Potential for changing existing guidance |
| Evidence is most confusing or controversial |  | Unsatisfactory guidance |
| There is currently uncertainty or inconsistency in practice |  | Uncertainty or controversy about best practice |
| Questions exist about screening, diagnosis and treatment |  | Uncertainty or controversy about best practice |

| **Reddy, 2014** | | |
| --- | --- | --- |
| **Authors’ prioritization criteria** |  | **Prioritization criteria reclassification** |
| Size of problem - individual |  | Health burden |
| Size of problem - society |  | Burden on the healthcare system |
| Size of problem - inequality |  | Equity relevance |
| Making a difference - feasibility |  | Potential for changing existing guidance; Feasibility of intervention implementation |
| Making a difference - range and fit with current guidance |  | Potential for changing existing guidance;  Unsatisfactory guidance |
| Making a difference - availability of evidence |  | Potential for changing existing guidance; Availability of evidence |
| Current variation in practice |  | Practice variation |

| **Mounesan, 2016** | | |
| --- | --- | --- |
| **Authors’ prioritization criteria** | **Authors’ definitions** | **Prioritization criteria reclassification** |
| Magnitude/frequency of the problem | Prevalence, Incidence, Burden of disease taking into account mortality and morbidity, QOL and fertility, Complexity of the problem at level of prevention (promote health) and patient level (patient with single/acute/multiple problems with social worries) | Health burden |
| Variation in problem management techniques | Variation in clinical practice in problem-solving | Practice variation |
| The capacity to improve health outcomes | Improvement of health outcomes, on the basis of the patient’s performance and experience taking into account effectiveness, efficiency and efficacy | Impact on health outcomes |
| The capacity to improve costs | Estimating the positive economic effects on the health system and society, taking into account cost and cost-efficiency | Economic impact |
| Significance of the main population affected by the CPG | Population groups: children working age adults, pregnant women, society’s vulnerable/low-income groups | Equity relevance |
| Risk capacity | The possible occurrence of serious side-effects of treatment, risks of using technology | Impact on health outcomes |
| Physicians' interest | Preferences of the professional community and high acceptability of the topic | Interest at the health professional level |
| The society's demands/worries | The population’s concern/high demand of the society | Interest at the consumer level |
| Necessity and urgency | National health plan (meeting national demands), national health priorities, the risk of waiting and postponing the problem, new found issues | Interest at the national level; Urgency |
| Need for evidence | The need for new information/modification of evidence, significance/added value of new evidence, lack of high-quality CPGs, need to domesticize CPGs, need to update national CPGs | Unsatisfactory guidance; Potential for changing existing guidance |
| Feasibility and applicability (system's capacity to implement) | The ease of developing recommendations and the feasibility to disseminate them, the socio-political feasibility, insurance and facilities, commitments and ethics, environmental health, human right (e.g. is the process politically doable and does it comply with governmental policies?) | Feasibility of intervention implementation |
| Persistence of the problem | Persistence of the problem for at least 3 years | Health burden |
