# Supplementary material for: Prioritization approaches in the development of health practice guidelines: a systematic review
Source: BMC Health Serv Res. 2019 Oct 15;19:692. doi: 10.1186/s12913-019-4567-2 (PMC6792189; doi:10.1186/s12913-019-4567-2)
Supplement: Supplementary file 4 — Additional file 4. Detailed findings of the included papers on the development processes of the prioritization approaches and the aspects to be addressed when prioritizing guideline topics. This represents a detailed tabular description of each of the 10 included prioritization approaches. [file 12913_2019_4567_MOESM4_ESM.docx]

**Appendix 4:** Detailed findings of the included papers on the development processes of the prioritization approaches and the aspects to be addressed when prioritizing guideline topics.

**Study ID: Battista, 1995**

This paper is the third of a series of six guidelines workshops that were prepared by the authors.

| **Steps of the development process of the approaches for prioritizing guideline topics** | |
| --- | --- |
| 1. Literature review (review of the U.S. literature and assessment of its applicability to Canadian context) | |
| Type of search | - Database search: Index Medicus |
|  | - Review of references of published material and files of Council for Health Technology Assessment in Quebec |
|  | - Consulting with knowledgeable individuals to check other sources of material on priority setting, however, none were identified |
| Time limit for search | 1985-1994 (Index Medicus) |
| 2. Stakeholders Involvement (survey) | |
| Selection method | potential workshop participants |
| Participants' type | physician licensing authorities |
|  | governments and para-governmental organizations |
|  | provincial and territorial medical associations |
|  | other organizations representing physicians at the provincial level |
|  | national specialty societies |
|  | others |
| Number of participants | 55 out of 107 (51.4%) |
| Addressed questions | - rating importance of 7 criteria using a 5-point scale from very important to not important at all |
|  | - respondents were not required to rank the criteria relative to each other |
|  | - average scores for each of the seven items were calculated for each group of participants |
|  | - suggesting other themes |
|  | - describing priority setting in the organizations they represent: little information about how priorities were actually set |
| 3. Identification of prioritization criteria | |
| Sources | - literature review |
|  | - survey results |
| Initial criteria list | 4 from the literature, 2 main and 2 associated criteria |
| Final criteria list | 10 criteria associated to 4 themes |
| **Aspects proposed to be addressed when prioritizing guideline topics (framework)** | |
| Aspects | **→ Identification and involvement of stakeholders** |
|  | - guideline developers should consider consulting with stakeholders before selecting topics for guidelines |
|  | - types: members of guideline developing organizations, potential end users, other stakeholders, as well as a careful consideration of a meaningful engagement of patients and the community |
|  | **→ Factors to consider when consulting stakeholders** |
|  | - feasibility of the topics given the resources of the developers |
|  | - conditions of practice into which the guidelines will be introduced |
|  | - likelihood that the guidelines will improve the health of the population and limit costs to the health care system |
|  | quantitative data should be incorporated when available |
|  | **→ Process documentation** |
|  | - documenting the process should be maintained and made available to members and other stakeholders |
|  | - documentation should form the basis for evaluating the guidelines development process |
| Potential challenges | **Data required in analytical priority-setting processes may be difficult or almost impossible to obtain for practice-related activity.** |

**Study ID: Field, 1995**

This is a report prepared by the Committee on Methods for Setting Priorities for Guidelines Development appointed by the Institute of Medicine in the United States.

| **Steps of the development process of the approaches for prioritizing guideline topics** | | |
| --- | --- | --- |
| 1. Formulation of a committee | | |
| Selection method | appointment by the IOM | |
| Participants' type | experts in guidelines development and implementation | |
|  | experts in health services research | |
|  | experts in health care delivery and health policy | |
| Number of members | 12 | |
| Process | the committee met twice, once in conjunction with an invitational workshop | |
| 2. Literature review | | |
| Type of search | | evaluation of priority setting processes used by other public and private organizations: staff from several organizations informed the committee about their priority setting processes |
| **Aspects proposed to be addressed when prioritizing guideline topics** | | |
| Aspects | **→ Involvement of stakeholders** | |
|  | - seeking experts' input via structured Delphi-like procedures or topic rankings by mail, fax, or electronic mail | |
|  | - surveys are to be designed using specific and explicit questions that are consistent with standard protocols for questionnaire construction | |
|  | - following more formal procedures to arrive at group judgments during priority setting meetings | |
|  | **→ Development of a basic procedure manual for priority-setting activities** | |
|  | **→ The committee recommended 6 general criteria to be applied in considering topics for guideline development** | |

**Study ID: McClarey, 1995**

The Royal College of Nursing (RCN) in the United Kingdom was working on developing national clinical guidelines as part of its clinical effectiveness initiative; two on pre-determined topics and one on a new one.

The aim was to develop multi-professional guidelines with focus on areas of care that nurses can assume the main responsibility and benefit the most from the clinical guideline.

| **Steps of the development process of the approaches for prioritizing guideline topics** | |
| --- | --- |
| 1. Review of existing methods and databases for prioritizing topics for clinical guideline development | |
| Type of search | The RCN model was developed from existing models in the literature |
| 2. Stakeholder involvement (convening of a multi-professional multi-agency clinical guidelines project group) | |
| Selection method | not applicable |
| Participants' type | representatives from professional organizations |
| Addressed issues | nursing policy analysts |
|  | researchers |
|  | patient/consumer representatives |
|  | review of existing guideline topics prioritization methods |
|  | consulting with guideline coordinators at other professional organizations |
| 3. Identification of prioritization criteria | |
| Sources | The identified criteria are based on criteria developed by the Clinical Outcomes Group (COG) of the National Health Service Executive (NHSE). |
| **Aspects proposed to be addressed when prioritizing guideline topics (RCN model)** | |
| Guiding principles for topic selection | - valuable to patients |
|  | - positively influence care quality and patient outcomes |
|  | - do not replicate clinical guidelines or similar forms of guidance that already exist |
|  | - fulfil criteria for guideline selection (see below for details) |
|  | - have been sufficiently researched to enable evidence-linked guidelines to be developed |
| Aspects | **→ The initial list is created by collecting data from RCN databases and professional groups and patient representatives.** |
|  | **→ The list is refined by grouping into themes** |
|  | **→ Prioritization criteria is applied to select the priority 10 topics** |
|  | **→ Search for existing guidelines and systematic reviews on the topics** |
|  | **→ Prioritization criteria is applied to select the priority topic** |
|  | **→ Topics are either considered for guideline development or feedback is shared with original collaborators for alternative action** |
| Limitations | - The model does not include a quantitative mechanism to rank or weight prioritization criteria |
|  | - The model's focus is on prioritizing topics at a national level in the U.K. which might not necessarily reflect sensitivity to local issues |
| Suggested improvements | The authors considered adding an element to the RCN model on the identification of local champions to assist in identifying topics for local guidelines. |

**Study ID: Oxman, 2006**

This paper is the second of a series of 16 reviews that were prepared by the World Health Organization (WHO) Advisory Committee as a background for advice for the WHO.

The overall aim of the series was to advise the WHO on how to use more rigorous processes to ensure that healthcare recommendations are informed by best available evidence.

The authors did not conduct a full systematic review.

| **Steps of the development process of the approaches for prioritizing guideline topics** | |
| --- | --- |
| 1. Literature review | |
| Type of search | - Database search: PubMed |
|  | - 3 databases of methodological studies: Cochrane Methodology Register, website of the 5th International Conference on Priorities in Health Care, and references that authors had in their files |
| 2. Identification of prioritization criteria | |
| Sources | - literature review |
|  | - authors' consideration of what is being done at the WHO and other organizations and aims and strategic advantage of the WHO |
|  | - authors' logical arguments |
| Final criteria list | - 5 criteria with explanation |
| Condition | The application of the identified criteria requires making judgements and this has to be made openly. |
| **Aspects proposed to be addressed when prioritizing guideline topics** | |
| Aspects | **→ The allocation of resources to the development of recommendations should be part of the routine budgeting process rather than a separate exercise.** |
|  | **→ Criteria for establishing priorities should be applied using a systematic and transparent process.** |
|  | **→ Because data to inform judgements are often lacking, unmeasured factors should also be considered – explicitly and transparently.** |
|  | **→ The process should include consultation with potential end users and other stakeholders, including the public, using well-constructed questions, and possibly using Delphi-like procedures.** |
|  | **→ Groups that include stakeholders and people with relevant types of expertise should make decisions. Group processes should ensure full participation by all members of the group.** |
|  | **→ The process used to select topics should be documented and open to inspection.** |
| Scope | - Both centralized and decentralized processes should be used within WHO across headquarters, regions and countries; and across different technical areas. |
|  | - Decentralized processes can be considered as separate "tracks". |
|  | - Separate tracks should be used for considering issues for specific areas, populations, conditions or concerns. The rationales for designating special tracks should be defined clearly; i.e. why they warrant special consideration. |
|  | - Updating of guidelines could also be considered as a separate "track", taking account of issues such as the need for corrections and the availability of new evidence. |

**Study ID: Ketola, 2007**

The board of the national guideline body in Finland (Current Care) realized the need to review their guideline topic selection process as the number of guidelines reached 50.

The board appointed a research team to draft a new version of the criteria for topic prioritization.

The aim was to develop a practical structured prioritization tool (PRIO) to assess guideline topics that have been suggested to the organization responsible for producing guidelines and assess its usefulness in the selection of Current Care topics in two samples of Current Care guidelines: some already published and some in development.

| **Steps of the development process of the approaches for prioritizing guideline topics** | | |
| --- | --- | --- |
| 1. Review of criteria used by guideline and health technology assessment organizations in other countries | | |
| Number of criteria | 22 | |
| 2. Stakeholder involvement (phone interviews) | | |
| Selection method | not applicable | |
| Participants' type | researchers | |
|  | members of the Current Care Board | |
|  | experts from health care organizations: Ministry of Social Affairs and Health representatives, hospital districts (secondary care) representatives, health centers (primary care) representatives | |
| Number of participants | 10 | |
| Type of interviews | phone interviews | |
| Questions | importance of the specified criteria and views of Current Care selection process | |
| Analysis | qualitative analysis | |
| 3. Identification of criteria and assigning weight | | |
| Sources | literature review and interview results | |
| Initial list | 22 | |
| Process | - 3 reviewers from the research group randomly selected 10 Current Care published guideline topics and 10 being published at the time of study (with their supplementary data) for evaluation | |
|  | - reviewers independently assessed the performance of the instrument on a random set of published guidelines | |
|  | - 2 reviewers then repeated the evaluation | |
|  | - disagreements were resolved by consensus | |
| Weighting of criteria | - importance | |
|  | - highlighting of the social and economic effects of the health problem | |
|  | - practice variation | |
| Final criteria list | - criteria were finalized in coordination with the board of Current Care | |
|  | - 8 final criteria listed, weighted and described criteria | |
| Additional comments | Other considerations should be taken into account when prioritizing guideline topics and developers cannot rely solely on a list of criteria. | |
| **Aspects proposed to be addressed when prioritizing guideline topics (PRIO-tool)** | | |
| Aspects | | **→ The need for new guidance emerges from professional societies or other sources** |
|  |  | **→ The PRIO tool (available on 'Current Care' website is used to suggest topics to the board** |
|  |  | **→ The board discusses the suggestions** |
|  |  | **→ In case approved:** |
|  |  | - editorial board schedules work on the received suggestions and then drafts an approval letter that describes next steps |
|  |  | - chairman and editor are nominated and the guideline group is recruited |
|  |  | - guideline group initiates the work (18-24 months) |
|  |  | **→ In case of no approval** |
|  |  | - reasons of rejection are explained in a letter |
| Implementation and evaluation | | Evaluation was done internally within the project by 3 independent reviewers |
|  |  | The guideline board will use the tool in future guideline development projects |
|  |  | The use of the tool in the prioritization and selection of guideline topics has to be re-evaluated after a few years. |

**Study ID: Reveiz, 2010**

The authors developed an instrument for Priority Determination of Topics (PDT) integrating quantitative and qualitative data from different sources.

| **Steps of the development process of the approaches for prioritizing guideline topics** | |
| --- | --- |
| 1. Literature review | |
| Type of search | - Database search: PubMed, Cochrane library, LILACS |
|  | - 8 databases related to guidelines: NICE, SIGN, WHO, G-I-N, the New Zealand Guidelines Group, the Canadian Medical Association InfoBase, Guidelines Advisory Committee, National Guideline Clearinghouse |
| Time limit for search | 1966-2008 (PubMed), 2008 (Cochrane library), 1982-2008 (LILACS) |
| 2. Identification of criteria | |
| Initial criteria list | 41 criteria under 10 domains |
| Scoring of criteria | - perceived importance of each of the 10 domains |
|  | - independent scoring by evaluators |
|  | - scale: 0 to 100 |
| Evaluation quality of evidence | score: good, moderate, bad, professional experience only, no information, not applicable |
|  |  |
| 3. Face validity | |
| Evaluators | epidemiologists |
|  | public health physician |
|  | psychologist |
|  | health administrator |
| Reflections | relevant instrument with a logical tie between its purpose and the criteria |
|  | clear, straightforward, reasonable, and grammatically correct questions and instructions |
|  | appropriate and well-defined choice options |
| 4. Stakeholder involvement (online survey) | |
| Selection method | convenience sampling |
|  | identification of participants from different sources (research institution lists, guideline developers and stakeholders from Colombia found in Google Scholar, colleagues etc.) and those who had an active electronic address |
| Participants' type | external stakeholders involved in clinical practice guideline development |
|  | end users of guidelines (patients, health care providers including clinical staff, government officials, representatives from the pharmaceutical industry and private health care managers and academic researchers) |
| Aim | to collect data to compare different rating procedures for the prioritization methodology |
| Number of participants | 60 out of 90 |
| Addressed questions | - the instrument with instructions was sent for rating each domain according to its relevance for PDT in CPGs (range 0 to 100) |
|  | - participants were also asked about additional domains or criteria and comments on the instrument |
|  | - 4 reminders |
| Main outcome | no substantial modifications to the instrument were suggested |
|  | results on weights for each domain were incorporated in the rating procedures |
| 5. Pilot-testing | |
| Number of participants | 38 |
| Participants' type | **thematic group** |
|  | - experts in the field |
|  | - methodological consultant |
|  | - expert representatives from the Medical School and from the Nursing School |
|  | **administrative group** |
|  | - expert in psychology |
|  | - members from the hospital board |
|  | - project manager |
| Process (workshop) | - thematic team suggested 3 to 5 clinical topics that could potentially be selected for developing a guideline |
|  | - participants used the instrument (see below) to score importance of domains for each proposed topic |
|  | - web-based tool was developed to allow participants to communicate and track the guideline development process |
|  | - participants rated the quality of the information used to support their judgments for each of the 41 items on the form |
|  | - rankings were categorized by team and topics were listed as having low, intermediate or high relevance and then a final selection was made during a consensus meeting |
|  |  |
| **Aspects proposed to be addressed when prioritizing guideline topics (instrument)** | |
| Aspects | **→ Involvement of stakeholders** |
|  | the contributions of various stakeholders is essential to prevent the possibility of a few stakeholders producing biased topics for guidelines |
|  | **→ Identification of initial list of topics** |
|  | experts in the field and s methodologists would suggest 3 to 5 clinical topics that could potentially be selected for developing a clinical practice guideline |
|  | **→ Instrument uses the criteria identified by the authors** |

**Study ID: Atkins, 2012**

This is the second of a series of 14 articles prepared by international methodologists and researchers in order to advise guideline developers in respiratory and other diseases.

The authors did not conduct a full or formal systematic review. This is an update of an existing priority setting review.

The authors' work is based on available evidence, experience in priority setting and working with guideline developers, reported organizational practice in developing guidelines, and workshop discussions.

| **Steps of the development process of the approaches for prioritizing guideline topics** | |
| --- | --- |
| 1. Literature review | |
| Type of search | - Database search: PubMed |
|  | - 3 databases of methodologic studies relating to guidelines, systematic reviews, and relevant methodological research: Cochrane Methodology Register, the U.S. National Guideline Clearinghouse, and the Guidelines International Network |
|  | - Consulting with references from the previous review and authors' own files. |
|  | - Review of guidelines as of September 2011 on COPD from major international organizations and examining whether they described their process for identifying topics for review and recommendation. |
| Time limit for search | 1990 to September, 2011 (PubMed) |
| 2. Identification of criteria | |
| List of criteria with their relevant sources of data based on literature findings | |
| **Aspects proposed to be addressed when prioritizing guideline topics** | |
| **Prioritization should be done at several steps of the guideline development process with several factors to be considered as described by the authors** | |
| Steps and factors | → To identify priorities for guideline development |
|  | resources, health burden, stakeholder input, data availability, practice variation |
|  | → To identify target audience and guideline's scope |
|  | clinicians' interest, patterns of care, quality gaps that can be improved;  the scope should address the target audience's most common problems |
|  | → To prioritize questions of potential interest |
|  | cost and epidemiology of disease, patterns of practice, stakeholder input;  priority should be based on high burden, evidence availability, controversy (ongoing), practice variation, and potential to improve practice |
|  | → To prioritize efforts for evidence synthesis |
|  | use high quality systematic reviews, new efforts should be oriented towards areas with the most complex or controversial evidence |
|  | → To prioritize recommendations |
|  | quality measures (feasibility and reliability of data collection and sample size);  recommendations with the most significant impact and best evidence while focusing on areas with existing quality gaps should be developed |
|  | → To prioritize recommendations for research |
|  | studies are feasible, address important gaps and could improve practice |
| Generation of initial list of topics | - clinicians, experts, and patients can be surveyed for candidate topics |
|  | - formal or informal processes (e.g. review of guidelines) to create a list of topics have been more commonly used |
|  | - clinical guidelines typically structure the specific content areas using pathophysiology of disease, elements of care, and epidemiology of disease |
|  | - formal processes are used to allow stakeholders to comment on scope and specific questions to be addressed by a systematic review or guideline |
|  | - “horizon” issues arising from emerging technologies and treatments should be identified by reviewing abstracts of major research meetings, editorials, and recent drug approvals |
| Stakeholder involvement in setting priorities | - various stakeholders and end users |
|  | - clinicians, professional organizations, policymakers, payers, government bodies, quality organizations, and patient representatives |
|  | - input on key questions is sought from various stakeholders including the industry and is being allowed by government-funded organizations |
|  | - research conducted by stakeholders should be assessed for inclusion in a guideline based on a transparent and independent assessment |

**Study ID: Schünemann, 2014**

The authors aimed to systematically develop a comprehensive checklist to be considered by guideline developers at all guideline stages; one of which is priority setting.

The authors also identify gaps in available tools and provide a way to fill the gaps. They provide resources (training materials) to assist guideline developers seeking to develop, implement, and update guidelines.

| **Steps of the development process of the approaches for prioritizing guideline topics** | |
| --- | --- |
| 1. Literature review | |
| Type of search | Database search (iterative process informed by systematic review) |
|  | methodology reports and guidelines for guidelines |
|  | guideline development manuals from governmental bodies |
|  | guideline development manuals from professional |
|  | The data abstraction forms used were pilot-tested |
| 2. Consultation with experts from the team | |
| Selection method | experts from the group who work in the field for over a decade, are members on key committees, and attend meetings in the field |
| Participants' type | experts |
|  | other stakeholders in guideline development in the group |
|  | researchers |
| Number of participants | 10 |
| Tasks | identification of additional resources to be included and necessary omissions |
|  | provision of feedback and suggestions about completeness of the checklist |
|  | review of documents to assess if saturation was reached and checklist is complete |
| **Aspects proposed to be addressed when prioritizing guideline topics (checklist: sections on priority setting)** | |
| Checklist | - The checklist is for guideline development and is organized into 18 topics with their corresponding items |
|  | - Two topics are related to prioritization and those are "Priority Setting" and "Identifying target audience and topic selection" |
| Aspects | **Priority Setting (topic 2 in the checklist)** |
|  | → deciding on a priority setting process and on who will direct it (priorities of sponsoring organization, or priorities referred by government or by professional societies) |
|  | → applying specific criteria in a systematic and transparent way in deciding on the guideline topic |
|  | → stakeholder involvement |
|  | → consider how different perspectives will be considered |
|  | → determine whether adaptation of an existing guideline is possible or if a new one is needed based on credibility assessment |
|  | → need for collaborative efforts in the development process |
|  | → do a scoping exercise to evaluate possible implementation issues and barriers |
|  | → select a consensus method |
|  | → documentation and transparency |
|  | **Identifying target audience and topic selection (topic 5 in the checklist)** |
|  | → use of criteria in prioritizing the guideline topic |
|  | → consultations to ensure all topics are covered and meet the needs of target audience |
|  | → select appropriate consensus method to agree on all final topics to be addressed |
|  | → documentation and transparency |
| Limitations | - equal weight for every step in the guideline development process |
|  | - areas that lack formal guidance and are not covered in guideline manuals and methodology reports may not be well represented in this checklist |

**Study ID: Reddy, 2014**

This study explores the role of the Analytic Hierarchy Process (AHP) in the prioritization process of the Centre for Public Health at the United Kingdom's National Institute for Health and Care Excellence (NICE)

| **Steps of the development process of the approaches for prioritizing guideline topics** | | |
| --- | --- | --- |
| 1. Formulation of a committee | | |
| Selection method | | - should not be current members of the NICE Topic Advisory Workshop (TAW) |
|  |  | - had worked on other NICE committees and were familiar with NICE processes |
|  |  | - volunteered to take part after being invited in a group email from a NICE representative |
| Participants' type | | facilitator |
|  |  | staff members from the Centre for Public Health |
|  |  | lay members of NICE's Public Health Interventions Advisory Committee (PHIAC) |
|  |  | public health experts |
|  |  | public health statistician |
| Number of members | | 9 |
| 2. Identification of criteria and assigning weight (using the AHP) | | |
| Sources | | stakeholders' input |
|  |  | each participant was asked to individually write 3 criteria and then share with the group to create clusters of relevant criteria |
| Initial criteria list | | 5 clusters of themes |
| Weighting of criteria | | - two criteria were selected and participants selected which was more important and by how much based on a specific scale |
|  |  | - the mean score was used to discuss and reach a consensus score |
|  |  | - the next pair of criteria was considered until all criteria were assessed and the relative importance of each criterion was derived |
| Final criteria list | | iterations were designed to remove redundant criteria |
|  |  | a hierarchy of 7 criteria |
| Condition | | discussion and judgement are necessary to ensure that priorities reflect the broader concerns of the health services, NICE and the public |
| 3. Pilot-testing (workshop) | | |
| Process | | the performance of topics was estimated on each criterion following a similar weighting pattern |
|  |  | a total score for each topic can thus be derived using a weighted sum approach |
|  |  | topics can be ranked and used for better informed discussion for future guidance to the Minister |
|  |  | sensitivity analysis was performed to finalize the ordering of topics |
| **Aspects proposed to be addressed when prioritizing guideline topics (AHP approach)** | | |
| Aspects | → AHP approach is one of the Multi-Criteria Decision Analysis (MCDA) techniques | |
|  | → explicit weighting of prioritization criteria and scores of proposed topics following the AHP scale represented below | |
|  | **Definition, weight and explanation** | |
|  | equal importance (1): two activities contribute equally to the objective | |
|  | moderate importance of one over another (3): experience and judgement strongly favor one activity over another | |
|  | essential or strong importance (5): experience and judgement strongly favor one activity over another | |
|  | very strong importance (7): an activity is strongly favored and its dominance demonstrated in practice | |
|  | extreme importance (9): the evidence favoring one | |
|  | intermediate values between two adjacent judgements (2,4,6,8): when compromise is needed | |
| Potential challenges | potential for increased gamesmanship | |
|  | - ministers might avoid the TAW process completely by fast tracking chosen topics | |
| Suggested improvements | - ensuring that experienced participants do not dominate discussions | |

**Study ID: Mounesan, 2016**

This study aimed to produce a manual for prioritization of clinical practice guidelines for family physicians. It was a multi-stage method using a qualitative approach.

| **Steps of the development process of the approaches for prioritizing guideline topics** | |
| --- | --- |
| 1. Literature review | |
| Type of search | Database search: PubMed, Google Scholar |
|  | 5 databases related to guidelines: SIGN, NICE, G-I-N, National Guideline Clearinghourse and other |
| Time limit for search | 2014 |
| 2. Stakeholders involvement (interviews) | |
| Selection method | purposeful sampling |
| Participants' type | service producers – providers and receivers |
|  | family physicians and specialties |
|  | policymakers familiar with the guideline development process |
| Number of participants | 10 |
| Addressed questions | - important priority setting criteria |
|  | - stakeholders |
|  | - issues involved in priority setting |
| Analysis | content analysis and member check |
| 3. Identification of criteria and determining criteria appropriateness | |
| Sources | literature review |
|  | interview results |
| Initial criteria list | 50 criteria: 40 criteria from literature review and 10 from interview results |
| Combined criteria list | 14 criteria with definitions |
|  | criteria definitions are based on available literature and expert opinion |
| Criteria appropriateness | Method: RAND/UCLA appropriateness method; two face-to-face rating rounds |
|  | rating range is 1 to 9: |
|  | - 1–3: inappropriate: criteria with median scores of 1–3 and with disagreement |
|  | - 4–6: uncertain: criteria with median scores of 4–6 and/or criteria whose medians fell in either score range but with disagreement |
|  | - 7–9: appropriate: criteria with median scores of 7–9 and those without disagreement |
| Final criteria list | List of 12 ranked criteria |
| 4. Preparing and piloting a prioritization manual | |
| Manual development | Based on qualitative findings and literature review |
| Manual sections | 5 manual sections and an ethical clause |
| Manual update | Pilot study |
| Pilot | Participants: nine experts and family physicians |
|  | Process: experts randomly selected 10 family physician guideline treatment topics that were primary priorities of Tehran University of Medical Sciences’ research centers for prioritization and ranking |
|  | Results: manual update |
|  | - a complementary point was added to the second section of the manual "Selection of important topics" |
|  | - a specific share was considered for emergency topics separately |
| **Aspects proposed to be addressed when prioritizing guideline topics (manual)** | |
| Aspects | → **Determining the levels of stewardship for the priority-setting process** |
|  | - national level |
|  | - peripheral level |
|  | → **Identifying important topics for the development of clinical guidelines for family physicians** |
|  | - informed by evidence including: scientific evidence, available reports, needs assessment and expert opinion |
|  | - priority setting is done separately for: prevention, diagnosis and treatment |
|  | → **Identification and involvement of stakeholders** |
|  | - selection method: stakeholder analysis technique |
|  | - range: 5-15 |
|  | - type: experienced family physicians and managers/directors |
|  | → **Application of well-defined criteria for rating and ranking important topics** |
|  | - rating of topics should be completed in face-to-face meetings and based on the 12 criteria |
|  | - topics are to be considered at the national and provincial levels |
|  | - 5-point Likert scale is to be used and the mean of the total score per criterion is to be considered |
|  | - discrepancies are to be resolved by discussion |
|  | → **Leadership and management of the priority-setting process** |
|  | - leadership should be well-defined |
|  | - 1 to 3 leaders |
|  | - the role is to steer, follow-up and provide feedback |
|  | **Ethical clause** |
|  | conflict of interest should be reported throughout the process |
